# *FLT-1* gene polymorphisms and protein expression profile in rheumatoid arthritis

**DOI:** 10.1371/journal.pone.0172018

**Published:** 2017-03-21

**Authors:** Agnieszka Paradowska-Gorycka, Anna Sowinska, Andrzej Pawlik, Damian Malinowski, Barbara Stypinska, Ewa Haladyj, Katarzyna Romanowska-Prochnicka, Marzena Olesinska

**Affiliations:** 1 Department of Biochemistry and Molecular Biology, National Institute of Geriatrics, Rheumatology and Rehabilitation, Warsaw, Poland; 2 Department of Computer Science and Statistics, Poznan University of Medical Sciences, Poznan, Poland; 3 Department of Physiology, Pomeranian Medical University, Szczecin, Poland; 4 Department of Pharmacology, Pomeranian Medical University, Szczecin, Poland; 5 Department of Connective Tissue Diseases, National Institute of Geriatrics, Rheumatology and Rehabilitation, Warsaw, Poland; 6 Department of Pathophysiology, Warsaw Medical University, Warsaw, Poland; Peking University First Hospital, CHINA

## Abstract

**Objectives:**

Inflammation and angiogenesis are a significant element of pathogenesis in rheumatoid arthritis (RA). The FLT-1- triggering factor for production of proinflammatory cytokines-might contributes to inflammation in patients with RA. Association of the *FLT-1* polymorphisms with different “angiogenic diseases” suggests that it may be a novel genetic risk factor also for RA. The aim of the study was to identify *FLT-1* genetic variants and their possible association with sFLT-1 levels, susceptibility to and severity of RA.

**Methods:**

The *FLT-1* gene polymorphisms were genotyped for 471 RA patients and 684 healthy individuals. Correlation analysis was performed with clinical parameters, cardiovascular disease (CVD) and anti-citrullinated peptide/protein antibody (ACPA) presence. The sFLT-1 serum levels were evaluated.

**Results:**

The *FLT-1* gene polymorphisms showed no significant differences in the proportion of cases and controls. Furthermore, the *FLT-1* rs2296188 T/C polymorphism was associated with ACPA-positive RA. Overall, rs9943922 T/C and rs2296283 G/A are in almost completed linkage disequilibrium (LD) with D’ = 0.97 and r2 = 0.83. The *FLT-1* rs7324510 A allele has shown association with VAS score (p = 0.035), DAS-28 score (p = 0.013) and ExRA presence (p = 0.027). Moreover, other clinical parameters were also higher in RA patients with this allele. In addition, *FLT-1* genetic variants conferred higher sFLT-1 levels in RA patients compared to controls.

**Conclusion:**

*FLT-1* rs7324510 C/A variant may be a new genetic risk factor for severity of RA. Examined factor highly predispose to more severe disease activity as well as higher sFLT-1 levels in RA.

## Introduction

Rheumatoid arthritis (RA) is the one of the most common, polygenic, autoimmune diseases. The main clinical feature of RA is destruction of cartilage and joint caused by inflammatory, erosive synovitis. Synovial membrane proliferation indicates that an intensive angiogenesis, occurs in the joints, is essential to promot and maintain RA as well as in the formation and growth of the synovial pannus [1, 2]. During RA, angiogenesis lead to the disease progression via increase the total vascular endothelial surface and enhanc the recruitment of leukocytes into the synovial tissue [2, 3]. The process of new vessels formation is regulated by many mediators, of which the central and the best characterized are vascular endothelial growth factor (VEGF) family and its receptors [4]. VEGF is a proangiogenicand angiogenic factor of physiological and pathogenic angiogenesis[5, 6]. Increased VEGF expression has been observed in synovial fluid and serum of RA patients and it showed correlation with C-reactive protein (CRP) as well as with radiological changes in the feet and hands. Quoted changes occur during the first year of the disease and, therefore, during the most intense angiogenesis. VEGF also plays a role as a functional bridge between inflammation and angiogenesis [7]. Moreover, VEGF interact with one or both of two tyrosine kinase receptors, VEGF receptor-1(VEGFR-1) and VEGF receptor-2 (VEGFR-2). While the biological function of the VEGFR-2 is understood, the VEGFR-1 remains largely elusive [8, 9]. We hypothesized that VEGFR-1also known as fms-related tyrosine kinase 1 (FLT-1), which triggering production of proinflammatory cytokines, might contribute to the inflammation in patients with RA. At least we know three main evidences to support this hypothesis. First, VEGFR-1 expression is not limited to the vascular endothelial cells. This expression induces abnormally high angiogenesis [8–10]. Second, VEGFR-1 has a central role in pathological angiogenesis during RA, mediated by not only VEGF, but also by placenta growth factor (PlGF) [11]. Third reason is the correlation between FLT-1 genetic variants and different angiogenic diseases suggesting that this mediator may represent the novel genetic risk factors for RA [11–15]. A better defining of the role of genetic factors and its clinical manifestation is necessary to identify individual’s susceptibility for development of RA and prevention of RA occurrence. In addition, an improved understanding of RA molecular pathogenesis will enable the development of new intervention strategies.

To confirm above hypothesis, we have assessed association between seven *FLT-1* single nucleotide polymorphisms (SNPs) located in both 3’UTR regions as well as introns and susceptibility to and severity of RA in the Polish population.

## Materials and methods

### Study population

The samples included in the present study were collected from 471 patients with RA and 684 healthy individuals. RA patients were recruited from the National Institute of Geriatrics, Rheumatology and Rehabilitation in Warsaw, Poland and Pomeranian Medical University in Szczecin, Poland. All the cases fulfilled the 1987 American College of Rheumatology (ACR) or the 2010 EULAR/ACR criteria for RA.

Controls (479 females and 205 males, age between 18 and 85 years) consisted of volunteers who have not shown any clinical or laboratory signs of autoimmune diseases. Patients and control subjects had the same socioeconomic status and were from the same geographical area. All subjects were of European ancestry. We selected a representative sample of the admixed urban Polish population.

Informed consent was obtained from all individual participants included in the study. The study was reviewed and approved by the Research Ethics Committee of the National Institute of Geriatrics, Rheumatology and Rehabilitation (of 29 May 2014), and by the Research Ethics Committee of the Pomeranian Medical University. All procedures performed in this study were in accordance with the ethical standards of our Institute and with the 1964 Helsinki declaration and its later amendments or comparable ethical standards.”

### Rheumatoid factor (RF) and anti-citrullinated peptide/protein antibody (ACPA) detection

The RF serum concentrations (34 IU ⁄ ml) were determined using the nefelometric method, whereas presence of second-generation ACPA (17 U⁄ ml) were detected using ELISA kits (Elecsys Anti-CCP assay; Roche Diagnostics GmbH, Basel, Switzerland) with measuring range 7– 500 U⁄ ml. Both theses parameters were determined only in RA patients.

### Genotyping

Peripheral blood (PB) was collected in vacutainers containing EDTA. Genomic DNA was extracted using the standard isothiocynate guanidine (GTC) extraction method and/or the QIAamp DNA Blood Mini Kit (Qiagen, Hilden, Germany). The TaqMan allelic discrimination assay was used to detect the genotypes of all examined polymorphisms (Applied Biosystems, Foster City, CA, USA). The reaction was performed in 10ul volume on StepOne Real-Time PCR system in RotorGene 6000 with the following amplification protocol: denaturation at 95°C for 10 min, followed by 40 cycles of denaturation at 92°C for 15 s, and annealing and extension at 60°C for 1 min. Negative controls and duplicate randomly selected samples were included to check the accuracy of genotyping.

### Soluble (s)FLT-1 protein measurements

Serum samples from RA patients and controls were separated from peripheral venous blood at room temperature and frozen at -86°C until analysis. sFLT-1 levels (ng/ml) in serum were measured with enzyme-linked immunosorbent assay kits (ELISA: Diaclone, Besancon Cedex, France), according to the manufacturer’s instructions. The limit of detection of human sFLT-1 was determined to be 0.03ng/ml. Each sample was assayed in duplicate and the intra-assay coefficient of variation was < 5.5%. Plates were read at an absorbance of 450 nm on LT-4000MS reader (Labtech International Ltd, Great Britain). Concentration was determined following a linear standard curve fit as per instruction’s recommendation.

### Statistical analysis

The results are presented as percentage for categorical variables, mean with 1 standard deviation for normally distributed continuous variables, or median (range) for non-normally distributed continuous variables as tested by the Shapiro–Wilk test. A P value of less than 0.05 was considered significant. Deviations from the Hardy-Weinberg Equilibrium (HWE) were tested for all examined SNPs using the Hardy Weinberg Simulator software (available at Institute of Human Genetics, Helmholtz Zentrum München, Germany). Linkage disequilibrium (LD) and coefficient (D′ and r2) for haplotypes and their frequencies were performed using the genetic statistical software SHEsis (http://analysis.bio-x.cn) [16, 17]. The Fisher exact probability test or chi-square test were used to evaluate differences in genotype and allele prevalence between the examined groups. Polymorphisms were tested for association using the chi-square test for trend (P trend). The Fisher exact test was used for power analysis. The association between SNPs and clinical/serological parameters were compared by the U Mann–Whitney test, t test, or Cochran–Cox test for continuous variables. Statistical analysis was performed using Graph-Pad InStat 3.10, 32 bit for Windows, created July 9, 2009 (GraphPad Software, Inc., San Diego, California, United States), CytelStudio version 10.0, created January 16, 2013 (CytelStudio Software Corporation, Cambridge, Massachusetts, United States), and Statistica version 10, 2011 (Stat Soft, Inc., Tulsa, Oklahoma, United States).

## Results

### Characteristics of the study cohort

Demographic and clinical as well as biochemical parameters of RA patients, summarized in Table 1, are collected at the time blood sampling. The median age of RA patients was 56 years; 71% were RF positive; and 82% had the ACPA antibodies. All included RA patients showed longstanding disease with disease duration > 10 years, DAS-28 score >5.18, HAQ score 1.625 and Larsen score 3. Evidence of coronary artery disease was found in 16% of patients; hypertension in 35%, and myocarditis in 3%; all this symptoms were classified as CVD. Comparing RA patients with CVD evens with those without CVD (Table 2) showed that patients with CVD were older (62 vs 54 yrs; p<0.0001) and had a higher activity of the disease. In those patients RF as well as ACPA were more frequent then in RA patients without CVD.

**Table 1:** Demographic and clinical characteristics of RA patients.

Characteristics	RA patients	
	N	mean values ±SD (range)
Age [years]	*471*	56 (22–89)
Disease duration[years]	*413*	10 (0–48)
Larsen	*453*	3 (0–5)
Number of swollen joints	*228*	3.5 (0–26)
Number of tender joints	*226*	8 (0–24)
ESR [mm/h]	*452*	30 (0–164)
CRP [mg/L]	*231*	13.7 (0–111)
Hemoglobin [g/dL]	*230*	12.7 (8.2–16.5)
VAS [mm]	*221*	56 (5–96)
DAS 28-CRP	*224*	5.18 (1.51–7.78)
HAQ	*193*	1.625 (0–3.125)
PLT [x10^3^/mm^3^]	*231*	301 (75–576)
Creatinine	*230*	0.7 (0–2.6)
	**N**	**n(%)**
RF presence	*319*	71
anti-CCP presence	*192*	82
Morning siffness	*213*	77
Organ symptoms	*114*	25
Coronary artery disease	*36*	16
Hypertension	*82*	35
Myocarditis	*8*	3

N – number of patients with clinical information; n- number of patients with positive clinical manifestation; DAS-28 - disease activity score for 28 joints, VAS - visual analogue scale (range 0–100), HAQ - Health Assessment Questionnaires (range 0–3), CRP - C-reactive protein, ESR - erythrocyte sedimentation ratio, PLT - platelet, RF - rheumatoid factor, anti-CCP - anti-CCP antibodies.

**Table 2:** Clinical characteristics RA patients with CVD.

parameter	patients with cardiovascular diseases		patients without cardiovascular diseases		p
	*N*	median (IQR)	*N*	median (IQR)	
age [years]	*103*	62 (14)	*131*	54 (13)	<0.0001
disease duration [years]	*89*	10 (11)	*102*	10 (11)	0.639
number of swollen joints	*99*	3 (7)	*129*	4 (7)	0.602
number of tender joints	*98*	8 (9)	*128*	8 (7.5)	0.384
Larsen	*102*	3 (1)	*131*	3 (1)	0.926
ESR [mm/h]	*102*	30 (30)	*131*	25 (26)	0.088
CRP [mg/L]	*99*	16 (32.2)	*131*	12 (27)	0.021
VAS [mm]	*97*	60 (41)	*124*	53.5 (38)	0.867
DAS-28	*97*	5.103 ± 1.33	*127*	4.959 ± 1.19	0.394
HAQ	*87*	1.625 (1.0)	*104*	1.5 (1.125)	0.122
Hb	*100*	12.95 (1.9)	*130*	12.7 (1.7)	0.692
PLT	*101*	307 (119)	*130*	297.5 (118)	0.788
Creatinine	*100*	0.7 (0.2)	*130*	0.675 (0.2)	0.100
parameter	patients with cardiovascular diseases		patients without cardiovascular diseases		p
	*N*	n (%)	*N*	n (%)	
women	*96*	93.2	*123*	94.6	0.863
RF +	*77*	75.5	*78*	61.9	0.029
anti-CCP +	*83*	82.2	*107*	81.7	0.922

N: no. patients with clinical information; n: no. patients with positive clinical manifestation; IQR: interquartile range

### *FLT-1* SNPs information and association of the individual SNPs with risk of RA

To verify the findings, randomly selected DNA samples were analyzed by direct sequencing using an ABI PRISM sequencer (Applied Biosystems, Foster City, USA) and the results were 100% in line. The genotyping success rate was greater than 94% in all cases. The MAF of the five chosen polymorphisms in our cohorts were similar to those in the Utah residents of northern and western European ancestry (HapMap database; S1 Table). Furthermore, the *FLT-1* minor rs2296188 C allele frequency was lower in Polish subjects (14-16%) than in other European populations (35%). Whereas, the *FLT-1* minor rs7337610 C allele frequency was higher in our cohort (60-62%) comparing to other European cohorts (49%). The all examined *FLT-1* polymorphisms genotype distributions were in Hardy-Weinberg equilibrium (HWE) in both RA patients and control group. Also, there was no evidence of any systematic bias in genotyping.

In the next step we investigated whether there was an association between selected *FLT-1* genetic variants and susceptibility to RA in our population (Table 3). There were no differences between the RA patients and healthy subjects in the genotype distribution as well as allele frequency of the seven SNPs (all p>0.2).

**Table 3:** Distribution of genotypes and allele frequencies of *FLT-1* SNPs among patients with RA and healthy subjects (p = RA vs controls).

***FLT-1* rs12858139 A/C**		RAn (%)	Controlsn (%)	OR (95% CI)	p*
	genotype				
Codominant	AA	111 (23.7)	159 (23.2)	-	-
	AC	217 (46.4)	320 (46.8)	0.971 (0.714–1.324)	0.907
	CC	140 (29.9)	205 (30.0)	0.978 (0.699–1.371)	0.959
Dominant	AA	111 (23.7)	159 (23.2)	-	-
	AC+CC	357 (76.3)	525 (76.8)	0.974 (0.732–1.299)	0.907
Recessive	AA+AC	32 (70.1)	479 (70.0)	-	-
	CC	140 (29.9)	205 (30.0)	10.22 (6.644–16.02)	0.920
Overdominant	AA+CC	251 (53.6)	364 (53.2)	-	-
	AC	217 (46.4)	320 (46.8)	0.983 (0.771–1.254)	0.937
	Alleles				
	A	439 (46.9)	638 (46.6)	-	-
	C	497 (53.1)	730 (53.4)	0.989 (0.835–1.173)	0.934
***FLT-1* rs2296188 T/C**		RAn (%)	Controlsn (%)	OR (95% CI)	p*
	genotype				
Codominant	TT	14 (3.0)	14 (2.1)	-	-
	TC	120 (25.8)	169 (24.7)	0.710 (0.302–1.674)	0.503
	CC	332 (71.2)	500 (73.2)	0.664 (0.289–1.525)	0.380
Dominant	TT	14 (3.0)	14 (2.1)	-	-
	TC+CC	452 (97.0)	669 (97.9)	0.676 (0.296–1.546)	0.402
Recessive	TT+TC	134 (28.8)	183 (26.8)	-	-
	CC	332 (71.2)	500 (73.2)	0.907 (0.692–1.190)	0.507
Overdominant	TT+CC	346 (74.3)	514 (75.3)	-	-
	TC	120 (25.8)	169 (24.7)	1.055 (0.796–1.395)	0.750
	Alleles				
	T	148 (15.9)	197 (14.4)	-	-
	C	784 (84.1)	1169 (85.6)	0.893 (0.704–1.134)	0.367
***FLT-1* rs9943922 T/C**		RAn (%)	Controlsn (%)	OR (95% CI)	p*
	genotype				
Codominant	TT	114 (24.3)	146 (21.4)	-	-
	TC	224 (47.9)	330 (48.3)	0.869 (0.639–1.185)	0.398
	CC	130 (27.8)	207 (30.3)	0.804 (0.571–1.133)	0.225
Dominant	TT	114 (24.4)	146 (21.4)	-	-
	TC+CC	354 (75.6)	537 (78.6)	0.844 (0.633–1.128)	0.264
Recessive	TT+TC	338 (72.2)	476 (69.7)	-	-
	CC	130 (27.8)	207 (30.3)	0.884 (0.676–1.156)	0.390
Overdominant	TT+CC	244 (52.1)	353 (51.7)	-	-
	TC	224 (47.9)	330 (48.3)	0.982 (0.771–1.251)	0.928
	Alleles				
	T	452 (48.3)	622 (45.5)	-	-
	C	484 (51.7)	744 (54.5)	0.895 (0.755–1.061)	0.208
***FLT-1* rs7324510 C/A**		RAn (%)	Controlsn (%)	OR (95% CI)	p*
	genotype				
Codominant	CC	15 (3.3)	26 (3.8)	-	-
	CA	122 (26.9)	203 (29.7)	1.042 (0.508–2.204)	1.000
	AA	316 (69.8)	455 (66.5)	1.204 (0.603–2.486)	0.699
Dominant	CC	15 (3.3)	26 (3.8)	-	-
	CA+AA	438 (96.7)	658 (96.2)	1.154 (0.581–2.372)	0.794
Recessive	CC+CA	137 (30.2)	229 (33.5)	-	-
	AA	316 (69.8)	455 (66.5)	1.161 (0.899–1.513)	0.281
Overdominant	CC+AA	331 (73.1)	481 (70.3)	-	-
	CA	122 (26.9)	203 (29.7)	0.873 (0.664–1.147)	0.349
	Alleles				
	C	152 (16.8)	255 (18.6)	-	-
	A	754 (83.2)	1113 (81.4)	1.137 (0.906–1.428)	0.280
***FLT-1* rs2296283 G/A**		RAn (%)	Controlsn (%)	OR (95% CI)	p*
	genotype				
Codominant	GG	103 (22.0)	139 (20.3)	-	-
	GA	229 (48.9)	325 (47.5)	0.951 (0.693–1.308)	0.806
	AA	136 (29.1)	220 (32.2)	0.834 (0.590–1.180)	0.326
Dominant	GG	103 (22.0)	139 (20.3)	-	-
	GA+AA	365 (78.0)	545 (79.7)	0.904 (0.672–1.219)	0.536
Recessive	GG+GA	332 (70.9)	464 (67.8)	-	-
	AA	136 (29.1)	220 (32.2)	0.864 (0.663–1.125)	0.291
Overdominant	GG+AA	239 (51.1)	359 (52.5)	-	-
	GA	229 (48.9)	325 (47.5)	1.058 (0.831–1.349)	0.680
	Alleles				
	G	435 (46.5)	603 (44.1)	-	-
	A	501 (53.5)	765 (55.9)	0.908 (0.766–1.077)	0.275
***FLT-1* rs3751397 A/T**		RAn (%)	Controlsn (%)	OR (95% CI)	p*
	genotype				
Codominant	AA	115 (25.8)	188 (27.5)	-	-
	AT	211 (47.3)	329 (48.1)	1.048 (0.777–1.416)	0.806
	TT	120 (26.9)	167 (24.4)	1.175 (0.833–1.656)	0.383
Dominant	AA	115 (25.8)	188 (27.5)	-	-
	AT+TT	331 (74.2)	496 (72.5)	1.091 (0.826–1.445)	0.575
Recessive	AA+AT	326 (73.1)	517 (75.6)	-	-
	TT	120 (26.9)	167 (24.4)	1.140 (0.859–1.509)	0.384
Overdominant	AA+TT	235 (52.7)	355 (51.9)	-	-
	AT	211 (47.3)	329 (48.1)	0.969 (0.757–1.239)	0.843
	Alleles				
	A	441 (49.4)	705 (51.5)	-	-
	T	451 (50.6)	663 (48.5)	1.087 (0.915–1.292)	0.352
***FLT-1* rs7337610 T/C**		RAn (%)	Controlsn (%)	OR (95% CI)	p*
	genotype				
Codominant	TT	84 (18.0)	104 (15.2)	-	-
	TC	208 (44.4)	306 (44.7)	0.842 (0.593–1.197)	0.359
	CC	176 (37.6)	274 (40.1)	0.795 (0.556–1.140)	0.224
Dominant	TT	84 (18.0)	104 (15.2)	-	-
	TC+CC	384 (82.0)	580 (84.8)	0.820 (0.591–1.139)	0.248
Recessive	TT+TC	292 (62.4)	410 (59.9)	-	-
	CC	176 (37.6)	274 (40.1)	0.902 (0.703–1.157)	0.438
Overdominant	TT+CC	260 (55.6)	378 (55.3)	-	-
	TC	208 (44.4)	306 (44.7)	0.988 (0.774–1.261)	0.970
	Alleles				
	T	376 (40.2)	514 (37.6)	-	-
	C	560 (59.8)	854 (62.4)	0.896 (0.753–1.067)	0.225

p* - χ^2^ test with Yate’ correction, p = RA vs controls, p≤0,05 was considered as significant.

### Association of *FLT-1* SNPs with CV events and ACPA presence in RA patients

To investigate association of *FLT-1* genetic variants with different subsets of RA, we defined RA subsets by the presence or absence of CV events as well as ACPA presence. We found that polymorphisms located in the *FLT-1* gene do not have influence on the cardiovascular events such as coronary artery disease (CAD), hypertension (HNT) and myocarditis (MI) in patients with RA (S2 Table). Furthermore, we found that one of the examined *FLT-1* gene polymorphisms at position rs2296188 T/C was associated with ACPA-positive RA (p = 0.03, S3 Table). Other *FLT-1* gene SNPs did not shown association with ACPA-positive or ACPA-negative RA in our population.

### Haplotypes of the *FLT-1* gene and risk of rheumatoid arthritis

**Fig 1:**
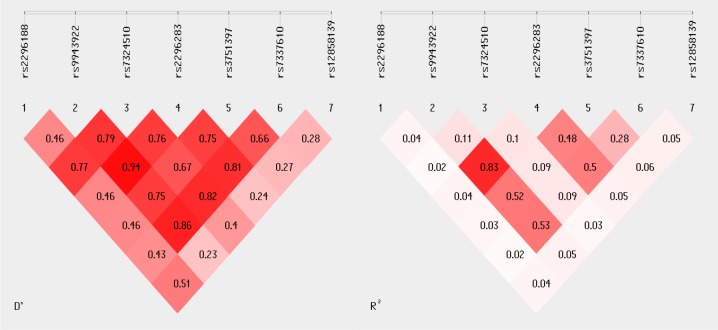
Linkage disequilibrium (LD) map of FLT-1 gene polymorphisms.

To further investigate whether *FLT-1* haplotypes have shown an association with RA, linkage disequilibrium (LD) map as well as haplotypes frequency differences was estimated for the seven defined FLT-1 gene polymorphisms. To create the haplotypes, *FLT-1* gene polymorphisms were analyzed in the following sequence: rs2296188, rs9943922, rs7324510, rs2296283, rs3751397, rs7337610, rs12858139. Nine potential *FLT-1* haplotypes were observed in both examined groups (S4 Table). Haplotypes with frequency <0.03 are ignored. The most frequent haplotype identified in our population was CCAAACA, which was observed in 16% of RA patients and 17% of controls. Very low frequencies (3%) in our cohort have shown CCAATCC haplotype. However, the frequency distribution of haplotypes was not significantly different between RA patients and healthy subjects. In this report the interaction between possible pair of SNPs visualized by the SHEsis program indicated that rs9943922 and rs2296283 are in almost completed LD with D’ = 0.97 and r^2^ = 0.83 (Fig 1). Moreover, the rs9943922, rs2296283 and rs7337610 polymorphisms had also very strong LD with D’>0.8 and r^2^ > 0.5. In contrast, of the *FLT-1* gene polymorphism at position rs12858139 had a very low LD with other SNPs with D’<0.5.

### *FLT-1* genetic variants and disease activity in RA patients

Although we found no association of the *FLT-1* gene polymorphisms with susceptibility to RA, we next analyzed whether *FLT-1* genetic variants have an impact on RA activity. We examined the genotype frequencies of associated SNPs, in different genetic models (dominant and recessive), according to demographic, clinical and laboratory parameters of RA. As shown in Table 4 the genotype-phenotype analysis showed significant correlation of the *FLT-1* rs7324510 C/A variant with VAS score (p = 0.035), DAS-28 score (p = 0.013) and ExRA presence (p = 0.027). All these parameter were higher in RA patients with rs7324510 A allele. Moreover, we also observed that carriers of this polymorphic allele (rs7324510 A) had a most of the clinical/biochemical parameters higher than RA patients with wild-type, rs7324510 C, allele.

**Table 4:** The disease activity and laboratory parameters in relations to *FLT-1* rs7324510 C/A.

**Parameter**	**CC**		**CA+AA**		p*
	**Mean±SD (median)**				
Age [years]	56 (22–84)		56 (22–89)		0.750
Disease duration [years]	6 (1–20)		10 (1–48)		0.144
Larsen	3 (1–4)		3 (0–5)		0.245
Number of swollen joints	3 (0–8)		4 (0–26)		0.572
Number of tender joints	4.5 (0–11)		8 (0–24)		0.287
ESR [mm/h]	26.5 (6–52)		30 (0–164)		0.315
CRP [mg/L]	6.7 (2.4–9)		13.7 (0–108.5)		0.124
Hemoglobin [g/dL]	13.55 ± 1.28		12.645 ± 1.40		0.203
VAS [mm]	29 (16–43)		54 (5–96)		0.035
DAS 28-CRP	3.433 ± 1.08		5.027 ± 1.26		0.013
HAQ	1.188 (0–1.375)		1.625 (0–3.125)		0.068
PLT [x10^3^/mm^3^]	328.5 (257–379)		308 (160–576)		0.740
Creatinine	0.7 (0.57–0.7)		0.68 (0–2.6)		0.906
	**CC**		**CA+AA**		p**
	***N***	**n (%)**	***N***	**n (%)**	
Women	*12*	85.7	*367*	88.2	0.893
RF presence	*9*	64.3	*281*	72	0.740
anti-CCP presence	*3*	75.0	*155*	83.3	0.815
Morning siffness	*4*	50.0	*167*	74.2	0.264
ExRA presence	*0*	0.0	*99*	24.9	0.027
Coronary artery disease	*0*	0.0	*31*	16.8	1.000

N – number of patients with clinical information; n- number of patients with positive clinical manifestation

p* - U Mann-Whitney test

p** - χ2 test; p < 0.05 was considered significant

Our analysis also demonstrated that rs12858139 A allele was specifically associated with morning stiffness (p = 0.03) and ExRA presence (p = 0.03), rs2296188 T allele with disease duration (p = 0.02) and rs3751397 T allele with mean value of ESR (p = 0.04) (data not shown). While the other examined *FLT-1* gene SNPs at position rs9943922, rs2296283, rs7337610 did not shown significant differences among RA patients divided according to disease activity and ExRA (data not shown).

### Correlation of the sFLT-1 protein level with susceptibility to and clinical phenotype of RA

To understand the possible role of FLT-1 in patients with rheumatoid arthritis we also determined the sFLT-1 expression levels in serum; it was assessed in 153 RA patients and 252 healthy subjects recruit from the genetic study cohort, by ELISA. As shown in S5 Table, the soluble FLT-1 expression levels was significantly increased in RA patients (0.106 ng/ml) compared with controls (0.096 ng/ml). Next we carried out a comparative analysis between sFLT-1 serum levels and clinical phenotype of RA (Table 5). Patients were divided into two groups: group I included the RA patients with the higher disease activity as well as RF-positive, ACPA-positive and CVD presence; whereas group II included the RA patients with the lowest disease activity without RF, ACPA and CVD. Association analysis did not show any significant relationship between sFLT-1 levels and clinical and biochemical parameters in our RA patients. We observed that sFLT-1 levels were higher in RA patients from the first group; patients with higher parameter of the diseases activity and joint damage; however, this association was not significant. Moreover, sFLT-1 showed a tendency to positively correlation with inflammatory marker – CRP. The sFLT-1 serum levels was higher in RA patients with CRP>13 (median: 0.113) compared to RA patients with CRP<13 (median 0.099, p = 0.065).

**Table 5:** Correlation of FLT-1 protein concentration of the various clinical parameters (RA).

Parameter		FLT-1 protein level			FLT-1protein level		p*
	*parameter group I*	*N*	median (IQR)	*parameter group II*	*N*	median (IQR)	
Age	age ≥ 56	*88*	0.112 (0.048)	age < 56	*51*	0.104 (0.058)	0.617
sex	women	*134*	0.110 (0.048)	men	*16*	0.095 (0.056)	0.416
RF	RF +	*74*	0.113 (0.053)	RF -	*36*	0.100 (0.042)	0.111
**anti-CCP**	ACPA +	*103*	0.112 (0.054)	ACPA -	*15*	0.094 (0.081)	0.294
disease duration	≥ 10	*31*	0.114 (0.085)	< 10	*42*	0.101 (0.043)	0.313
ESR	≥ 30	*48*	0.114 (0.057)	< 30	*68*	0.106 (0.049)	0.396
number of tender joints	≥ 7	*46*	0.113 (0.043)	< 7	*65*	0.106 (0.065)	0.691
number of swollen joints	≥ 3	*63*	0.114 (0.050)	< 3	*49*	0.101 (0.058)	0.207
CRP	≥ 13	*72*	0.113 (0.051)	< 13	*45*	0.099 (0.052)	0.065
DAS-28	≥ 5.0	*63*	0.113 (0.047)	< 5.0	*47*	0.103 (0.077)	0.574
HAQ	≥ 1.5	*48*	0.113 (0.050)	< 1.5	*37*	0.103 (0.048)	0.428
**cardiovascular diseases (CAD, HNT, MI)**	CVD +	*39*	0.114 (0.077)	CVD -	*77*	0.105 (0.045)	0.181

p* – Mann Whitney; p < 0.05 was considered significant

### Impact of *FLT-1* genetic variants on its serum levels

**Fig 2:**
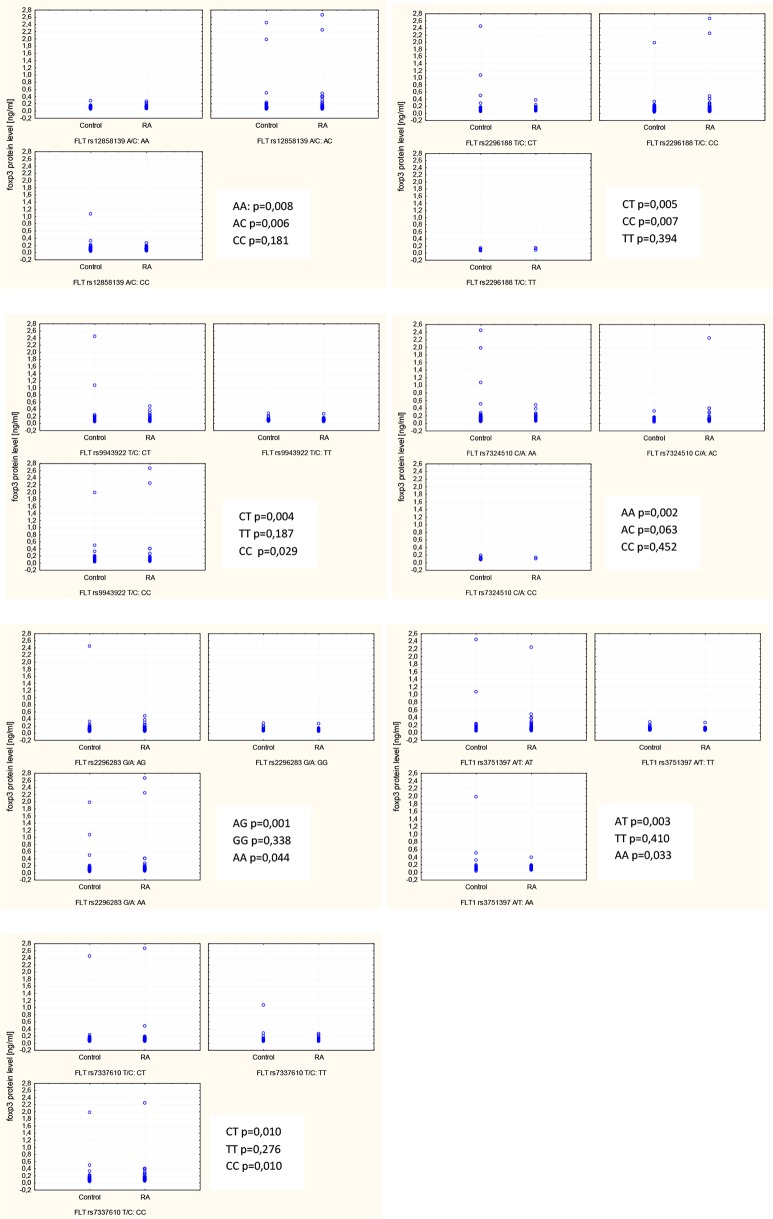
Variation in sFLT-1 serum levels in RA patients and controls in relation to FLT-1 gene polymorphisms.

Finally, we evaluated whether the presence of SNPs in the *FLT-1* gene may had an impact on its expression in serum. This analysis demonstrated that there is significant interaction between sFLT-1 levels and seven *FLT-1* genetic variants. Increased serum levels of sFLT-1 was observed in RA patients with rs12858139 AA and AC, rs2296188 CT and CC, rs9943922 CT and CC, rs7324510 AA, rs2296283 AG and AA, rs3751397 AT and AA as well as rs7337610 CT and CC genotypes compared to controls (Fig 2). In carriers of the other *FLT-1* genotypes serum sFLT-1 levels were not significantly different from those detected in the sera of healthy donors with the same genotypes.

## Discussion

In this study, for the first time, we demonstrated that *FLT-1* genetic variants may be associated with severity of RA as well as sFLT-1 serum levels in the Polish population. Further, we showed that *FLT-1* rs7324510 C/A polymorphism may be a new genetic risk factor for more severe disease activity in our patients. We observed that carriers of polymorphic rs7324510 A allele had a higher disease activity as well as FLT-1 serum levels than RA patients with wild-type rs7324510 C allele. Furthermore, we have also shown that *FLT-1* rs2296188 T/C variant may be a one of the genetic risk factor for ACPA-positive RA. Furthermore, we did not observed correlation between *FLT-1* gene SNPs and cardiovascular events in our patients.

Inflammation and angiogenesisplay a central role in many autoimmune diseases including RA. One of the most important mediators link both these processes are angiogenic/proangiogenic factors that stimulates angiogenesis as well as inflammation in rheumatoid joints.[18]. In patients with RA, FLT-1 expression is upregulated in the plasma, synovial fluid and synovium [18–20] and correlated with inflammatory markers such as ESR or CRP, what is in agreement with our findings. We also demonstrated that FLT-1 serum levels in RA patients were significantly higher than in controls, reflecting the angiogenesis and/or chronic inflammation in rheumatoid arthritis patients and trying to keep it under control. FLT-1 expression in arthritis is stimulated by two the most important proinflammatory cytokines - IL-1β and TNF-α, which can mediate the progression of bone destruction [18, 21, 22]. Furthermore, up-regulated FLT-1 expressions positively correlated with VEGF and PlGF concentration (data not shown).It leads to PlGF hyper-responsiveness and increase production of proinflammatory cytokines specific to RA [18]. Moreover, antibody against FLT-1 suppressed angiogenesis and inflammatory joint destruction in an animal model of RA [23]. There is the idea that blocking of FLT-1 could serve as an perfect target for selective reduction of both pathological angiogenesis and inflammatory reaction in active RA [5, 18].

Considering the important role of the FLT-1 in the modulation of angiogenesis and inflammatory processes as well as differences in genetic predispositions between populations we decided to carry out an analysis of selected *FLT-1* gene polymorphisms in relation to rheumatoid arthritis. Differences in the frequency of genotypes between research may be clarified by heterogeneity of the studied diseases and a variety ethnicities, as well as the limited size of the sample. Regulatory, structural or quantitative polymorphisms at the *FLT-1* locus may disrupt VEGF/PlGF signaling pathway and contribute to the susceptibility to some angiogenic conditions. Most of studied polymorphisms appear within the sequence of functional domains. However, an interesting group of polymorphisms are found in non-coding regions of the gene. In presented study we chose seven SNPs of which three are located in the functional 3’-UTR region (rs3751397, rs2296283, rs7337610) and four within introns (rs7324510, rs9943922, rs2296188, rs12858139). SNPs located in the 3’-UTR region of the gene may clash with mRNA translation and stability through effects on polyadenylation and regulatory miRNA-mRNA and protein-mRNA interactions, thus affecting the level of protein expression. However, this must be verified in the further research. Furthermore, the functional significance of SNPs located in noncoding region of *FLT-1* (introns) in unclear. We postulate that genetic variants located in an intronic region of the gene may affect the normal splicing and lead to increased transcriptional activity of this factor. Other studies have also demonstrated that noncoding polymorphisms may alter silencer or enhancer regions as well as may be markers for tagged mutations [11]. Although the results of our study showed no significant difference between RA patients and controls in genotype distribution and allele frequencies for all analyzed polymorphisms; however, we found that genetic variants in this gene may correlated with severity of RA in our Polish population. In the present study we have identified one polymorphisms rs7324510 C/A, located within introns of the *FLT-1* gene, significantly associated with more severe course of RA. We observed that rs7324510 A allele can affect the deterioration of the disease. The carriers of this allele have a significantly higher DAS-28, feel stronger pain assessed by VAS, have more frequent extra-articular symptoms and also achieved a higher score on the questionnaire assessing health (HAQ). In addition, in these RA patients other clinical/biochemical parameters such as number of tender/swollen joints or mean value of CRP and ESR were higher compared to RA patients with rs7324510 C allele. These results suggested that this genetic variant may affect the activity of *FLT-1* gene; however, the mechanism of how these polymorphisms influence the activity of FLT-1 is unknown.

In the present study, we also found evidence for association of the *FLT-1* polymorphisms with different regulation of sFLT-1 protein expression between RA patients and controls (Fig 2). Specifically, there were increased sFLT-1 serum levels in RA patients who had one or two copies of the risk alleles. In case of *FLT-1* rs7324510 C/A polymorphism, which was associated with disease activity in our study, we observed that RA patients with rs7324510 AA genotype had a higher sFLT-1 protein expression than controls.

These results of our study may suggest that *FLT-1* rs7324510 C/A genetic variant might be of biological significance as it contributes to sFLT-1 serum levels as well as severity of disease. However, larger population-based studies in different ethnic groups will be needed to validate the effects of genetic polymorphisms in the *FLT-1* gene on RA susceptibility and severity. Moreover, we believe that our results may be helpful to clarify the role of this angiogenic factor in the pathogenesis of RA. Furthermore, FLT-1 may be a candidate factor consistent with the severity of disease.

## Supporting information

S1 Table SNPs information and genotyping results for RA patients and control group. (DOC)

S2 Table RA patients with cardiovascular diseases (CAD, HNT, MI) in relation to FLT1 gene polymorphisms. (DOC)

S3 Table Distribution of genotypes and allele frequencies of FLT1 among patients with ACPA + and ACPA- (p = ACPA+ vs ACPA-). (DOC)

S4 Table Haplotype analysis for FLT-1 seven SNPs in RA patients and controls. (DOC)

S5 Table FLT1 protein level. (DOC)
