# Supplementary material for: FLT-1 gene polymorphisms and protein expression profile in rheumatoid arthritis
Source: PLoS One. 2017 Mar 21;12(3):e0172018. doi: 10.1371/journal.pone.0172018 (PMC5360214; doi:10.1371/journal.pone.0172018)
Supplement: S1 Table — (DOC) [file pone.0172018.s001.doc]

**S1 Table** . SNPs information and genotyping results for RA patients and control group.

| **SNP ID** | **Allele** | **SNP Type** | **MAF** | | | **p (HWE)** | |
| --- | --- | --- | --- | --- | --- | --- | --- |
|  |  |  | **RA** | **Control** | **HapMap-CEU** | **RA** | **Control** |
| **rs3751397** | A/T | 3’UTR | 0.50 | 0.48 | 0.41 | 0.26 | 0.33 |
| **rs7324510** | C/A | Intron | 0.83 | 0.81 | 0.76 | 0.45 | 0.57 |
| **rs9943922** | T/C | Intron | 0.52 | 0.54 | 0.41 | 0.37 | 0.5 |
| **rs2296283** | G/A | 3’-UTR | 0.53 | 0.56 | 0.41 | 0.72 | 0.34 |
| **rs2296188** | T/C | Intron | 0.16 | 0.14 | 0.35 | 0.43 | 0.95 |
| **rs12858139** | **A/C** | Intron | 0.53 | 0.53 | 0.46 | 0.14 | 0.12 |
| **rs7337610** | **T/C** | 3’-UTR | 0.60 | 0.62 | 0.49 | 0.1 | 0.23 |

MAF - minor allele frequency; HWE - Hardy-Weinberg equilibrium; CEU - Utah residents of northern and western European ancestry
