# Supplementary material for: FLT-1 gene polymorphisms and protein expression profile in rheumatoid arthritis
Source: PLoS One. 2017 Mar 21;12(3):e0172018. doi: 10.1371/journal.pone.0172018 (PMC5360214; doi:10.1371/journal.pone.0172018)
Supplement: S2 Table — (DOC) [file pone.0172018.s002.doc]

**S2 Table.** RA patients with cardiovascular diseases (CAD, HNT, MI) in relation to *FLT1* gene polymorphisms

| **Genotype** | **Patients with cardiovascular diseases** | | **Patients without cardiovascular diseases** | | **OR CI** | **p** |
| --- | --- | --- | --- | --- | --- | --- |
|  | **n** | **(%)** | **n** | **(%)** |  |  |
| **FLT rs12858139 A/C** |  |  |  |  |  |  |
| AA | *22* | 24.2 | *26* | 23.9 | - | - |
| AC | *40* | 43.9 | *46* | 42.2 | 1.028 (0.477 - 2.222) | 1.000 |
| CC | *29* | 31.9 | *37* | 33.9 | 0.926 (0.411 - 2.094) | 0.991 |
| **FLT rs2296188 T/C** |  |  |  |  |  |  |
| TT | *2* | 2.2 | *6* | 5.5 | - | - |
| TC | *25* | 27.8 | *24* | 22.0 | 3.125 (0.486 – 33.97) | 0.326 |
| CC | *63* | 70.0 | *79* | 72.5 | 2.392 (0.408 – 24.89) | 0.486 |
| **FLT rs9943922 T/C** |  |  |  |  |  |  |
| TT | *28* | 30.8 | *27* | 24.8 | - | - |
| TC | *35* | 38.4 | *46* | 42.2 | 0.734 (0.349 – 1.545) | 0.479 |
| CC | *28* | 30.8 | *36* | 33.0 | 0.750 (0.342 – 1.645) | 0.551 |
| **FLT rs7324510 C/A** |  |  |  |  |  |  |
| CC | *1* | 1.1 | *3* | 2.9 | - | - |
| CA | *23* | 25.9 | *33* | 32.7 | 2.091 (0.155 – 114.5) | 0.944 |
| AA | *65* | 73.0 | *65* | 64.4 | 3.00 (0.232 – 159.9) | 0.642 |
| **FLT rs2296283 G/A** |  |  |  |  |  |  |
| GG | *21* | 23.1 | *21* | 19.3 | - | - |
| GA | *45* | 49.4 | *57* | 52.3 | 0.789 (0.361 – 1.730) | 0.645 |
| AA | *25* | 27.5 | *31* | 28.4 | 0.807 (0.335 – 1.941) | 0.748 |
| **FLT1 rs3751397 A/T** |  |  |  |  |  |  |
| AA | *24* | 27.9 | *28* | 27.7 | - | - |
| AT | *33* | 38.4 | *47* | 46.6 | 0.819 (0.382 – 1.761) | 0.706 |
| TT | *29* | 33.7 | *26* | 25.7 | 1.301 (0.569 – 2.982) | 0.627 |
| **FLT rs7337610 T/C** |  |  |  |  |  |  |
| TT | *16* | 17.6 | *19* | 17.4 | - | - |
| TC | *33* | 36.3 | *37* | 34.0 | 1.059 (0.435 – 2.597) | 1.000 |
| CC | *42* | 46.1 | *53* | 48.6 | 0.941 (0.403 – 2.219) | 1.000 |

p - χ^2^ test with Yate’ correction, p = CVD+ vs CVD -, p≤0,05 was considered as significant
