# Supplementary material for: FLT-1 gene polymorphisms and protein expression profile in rheumatoid arthritis
Source: PLoS One. 2017 Mar 21;12(3):e0172018. doi: 10.1371/journal.pone.0172018 (PMC5360214; doi:10.1371/journal.pone.0172018)
Supplement: S3 Table — (DOC) [file pone.0172018.s003.doc]

**S3 Table.** Distribution of genotypes and allele frequencies of FLT1 among patients with ACPA + and ACPA- (p = ACPA+ vs ACPA-)

| **FLT rs12858139 A/C** |  | ACPA+  n (%) | ACPA-  n (%)* | OR (95% CI) | p value |
| --- | --- | --- | --- | --- | --- |
|  | genotype |  |  |  |  |
| Codominant | AA | 41 (24,7) | 7 (20,6) | - | - |
|  | AC | 72 (43,4) | 13 (38,2) | 0,946 (0,295 – 2,799) | 1,000 |
|  | CC | 53 (31,9) | 14 (41,2) | 0,646 (0,202 – 1,909) | 0,540 |
|  | Alleles |  |  |  |  |
|  | A | 154 (46,4) | 27 (39,7) | - | - |
|  | C | 178 (53,6) | 41 (60,3) | 0,761 (0,429 – 1,335) | 0,382 |
| **FLT rs2296188 T/C** |  | ACPA+  n (%) | ACPA-  n (%)* | OR (95% CI) | p value |
|  | genotype |  |  |  |  |
| Codominant | TT | 5 (3,0) | 3 (8,8) | - | - |
|  | TC | 40 (24,1) | 10 (29,4) | 2,4 (0,314 – 14,71) | 0,497 |
|  | CC | 121 (72,9) | 21 (61,8) | 3,457 (0,494 – 19,14) | 0,234 |
|  | Alleles |  |  |  |  |
|  | T | 50 (15,1) | 16 (28,1) | - | - |
|  | C | 282 (84,9) | 41 (71,9) | 2,201 (1,066 – 4,372) | 0,033 |
| **FLT rs9943922 T/C** |  | ACPA+  n (%) | ACPA-  n (%)* | OR (95% CI) | p value |
|  | genotype |  |  |  |  |
| Codominant | TT | 50 (30,1) | 6 (17,7) | - | - |
|  | TC | 64 (38,6) | 17 (50,0) | 0,452 (0,136 – 1,32) | 0,174 |
|  | CC | 52 (31,3) | 11 (32,3) | 0,567 (0,160 – 1,834) | 0,433 |
|  | Alleles |  |  |  |  |
|  | T | 164 (49,4) | 29 (42,6) | - | - |
|  | C | 168 (50,6) | 39 (57,4) | 0,762 (0,433 – 1,331) | 0,378 |
| **FLT rs7324510 C/A** |  | ACPA+  n (%) | ACPA-  n (%)* | OR (95% CI) | p value |
|  | genotype |  |  |  |  |
| Codominant | CC | 3 (1,9) | 1 (3,1) | - | - |
|  | CA | 48 (30,4) | 8 (25,0) | 2 (0,034 – 28,41) | 0,975 |
|  | AA | 107 (67,7) | 23 (71,9) | 1,551 (0,028 – 20,25) | 1,000 |
|  | Alleles |  |  |  |  |
|  | C | 54 (17,1) | 10 (15,6) | - | - |
|  | A | 262 (82,9) | 54 (84,4) | 0,899 (0,384 – 1,929) | 0,940 |
| **FLT rs2296283 G/A** |  | ACPA+  n (%) | ACPA-  n (%)* | OR (95% CI) | p value |
|  | genotype |  |  |  |  |
| Codominant | GG | 38 (22,9) | 5 (14,7) | - | - |
|  | GA | 84 (50,6) | 19 (55,9) | 0,582 (0,158 – 1,778) | 0,449 |
|  | AA | 44 (26,5) | 10 (29,4) | 0,579 (0,143 – 2,071) | 0,520 |
|  | Alleles |  |  |  |  |
|  | G | 160 (48,2) | 29 (42,6) | - | - |
|  | A | 172 (51,8) | 39 (57,4) | 0,799 (0,454 – 1,397) | 0,484 |
| **FLT1 rs3751397 A/T** |  | ACPA+  n (%) | ACPA-  n (%)* | OR (95% CI) | p value |
|  | genotype |  |  |  |  |
| Codominant | AA | 43 (27,9) | 8 (24,2) | - | - |
|  | AT | 62 (40,3) | 19 (57,6) | 0,607 (0,211 – 1,622) | 0,394 |
|  | TT | 49 (31,8) | 6 (18,2) | 1,426 (0,395 – 5,409) | 0,745 |
|  | Alleles |  |  |  |  |
|  | A | 148 (48,1) | 35 (53,0) | - | - |
|  | T | 160 (51,9) | 31 (47,0) | 1,221 (0,692 – 2,159) | 0,549 |
| **FLT rs7337610 T/C** |  | RA  n (%) | Controls  n (%)** | OR (95% CI) | p value |
|  | genotype |  |  |  |  |
| Codominant | TT | 32 (19,3) | 3 (8,8) | - | - |
|  | TC | 58 (34,9) | 13 (38,2) | 0,418 (0,072 – 1,695) | 0,303 |
|  | CC | 76 (45,8) | 18 (53,0) | 0,396 (0,070 – 1,507) | 0,233 |
|  | Alleles |  |  |  |  |
|  | T | 122 (36,7) | 19 (27,9) | - | - |
|  | C | 210 (63,3) | 49 (72,1) | 0,667 (0,354 – 1,218) | 0,211 |

p - χ2 test with Yate’ correction, p = ACPA + vs ACPA-, p≤0,05 was considered as significant
