# Supplementary material for: FLT-1 gene polymorphisms and protein expression profile in rheumatoid arthritis
Source: PLoS One. 2017 Mar 21;12(3):e0172018. doi: 10.1371/journal.pone.0172018 (PMC5360214; doi:10.1371/journal.pone.0172018)
Supplement: S4 Table — (DOC) [file pone.0172018.s004.doc]

**S4 Table .** Haplotype analysis for FLT-1 seven SNPs in RA patients and controls.

| **Haplotype** | **RA 2n= 1120** | **Control 2n=1362** | **OR [95% CI]** | **p*** |
| --- | --- | --- | --- | --- |
| CCAAACA | 183(0.163) | 234(0.171) | 0.943 [0.763~1.165] | 0.627 |
| CTAGTTC | 130(0.116) | 169(0.123) | 0.928 [0.727~1.184] | 0.577 |
| CTAGTTA | 130(0.116) | 151(0.11) | 1.054 [0.822~1.353] | 0.702 |
| CCAAACC | 102(0.091) | 134(0.098) | 0.919 [0.701~1.205] | 0.582 |
| CCCAACC | 82(0.073) | 105(0.076) | 0.947 [0.701~1.279] | 0.76 |
| CTAGTCC | 52(0.046) | 46(0.033) | 1.395 [0.93~2.091] | 0.12 |
| CCCAACA | 58(0.051) | 81(0.059) | 0.865 [0.611~1.223] | 0.43 |
| TTAGTTC | 46(0.041) | 65(0.047) | 0.855 [0.581~1.259] | 0.437 |
| CCAATCC | 38(0.033) | 47(0.034) | 0.984 [0.636~1.52] | 1 |

p*Fisher’s test; p < 0.05 was considered significant;
