# Supplementary material for: FLT-1 gene polymorphisms and protein expression profile in rheumatoid arthritis
Source: PLoS One. 2017 Mar 21;12(3):e0172018. doi: 10.1371/journal.pone.0172018 (PMC5360214; doi:10.1371/journal.pone.0172018)
Supplement: S5 Table — (DOC) [file pone.0172018.s005.doc]

**S5 Table**. FLT1 protein level

| Parameter | **RA group** | | **control group** | | p* |
| --- | --- | --- | --- | --- | --- |
|  | *N* | median (min-max; IQR) | *N* | Median (min-max; IQR) |  |
| protein level [ng/ml] | *153* | 0.106 (0.452 – 2.664; 0.047) | *252* | 0.096 (0.029 – 2.448; 0.026) | 0.0001 |

p* - MannWhitney; p < 0.05 was considered significant;
